# Pearl Millet: A Climate-Resilient Nutricereal for Mitigating Hidden Hunger and Provide Nutritional Security

**DOI:** 10.3389/fpls.2021.659938

**Published:** 2021-09-13

**Authors:** C. Tara Satyavathi, Supriya Ambawat, Vikas Khandelwal, Rakesh K. Srivastava

**Affiliations:** ^1^Indian Council of Agricultural Research - All India Coordinated Research Project on Pearl Millet, Jodhpur, India; ^2^Department of Molecular Breeding (Genomics Trait Discovery), International Crops Research Institute for Semi-arid Tropics, Patancheru, India

**Keywords:** pearl millet, climate-resilience, drought tolerance, abiotic stress, nutritional security

## Abstract

Pearl millet [*Pennisetum glaucum* (L.) R. Br.] is the sixth most important cereal crop after rice, wheat, maize, barley and sorghum. It is widely grown on 30 million ha in the arid and semi-arid tropical regions of Asia and Africa, accounting for almost half of the global millet production. Climate change affects crop production by directly influencing biophysical factors such as plant and animal growth along with the various areas associated with food processing and distribution. Assessment of the effects of global climate changes on agriculture can be helpful to anticipate and adapt farming to maximize the agricultural production more effectively. Pearl millet being a climate-resilient crop is important to minimize the adverse effects of climate change and has the potential to increase income and food security of farming communities in arid regions. Pearl millet has a deep root system and can survive in a wide range of ecological conditions under water scarcity. It has high photosynthetic efficiency with an excellent productivity and growth in low nutrient soil conditions and is less reliant on chemical fertilizers. These attributes have made it a crop of choice for cultivation in arid and semi-arid regions of the world; however, fewer efforts have been made to study the climate-resilient features of pearl millet in comparison to the other major cereals. Several hybrids and varieties of pearl millet were developed during the past 50 years in India by both the public and private sectors. Pearl millet is also nutritionally superior and rich in micronutrients such as iron and zinc and can mitigate malnutrition and hidden hunger. Inclusion of minimum standards for micronutrients—grain iron and zinc content in the cultivar release policy—is the first of its kind step taken in pearl millet anywhere in the world, which can lead toward enhanced food and nutritional security. The availability of high-quality whole-genome sequencing and re-sequencing information of several lines may aid genomic dissection of stress tolerance and provide a good opportunity to further exploit the nutritional and climate-resilient attributes of pearl millet. Hence, more efforts should be put into its genetic enhancement and improvement in inheritance to exploit it in a better way. Thus, pearl millet is the next-generation crop holding the potential of nutritional richness and the climate resilience and efforts must be targeted to develop nutritionally dense hybrids/varieties tolerant to drought using different omics approaches.

## Introduction

The changing climate is leading to an increase in global average temperature affecting agricultural production worldwide. Further, it directly influences biophysical factors such as plant and animal growth along with the different areas associated with food processing and distribution. Assessment of effects of global climate changes and deployment of new tools and strategies to mitigate their effect is crucial to maximizing agricultural production to meet out food demands of the increasing population. In this context, pearl millet is most useful as it is a nutritious, climate change-ready crop with enormous potential for yielding higher economic returns in marginal conditions in comparison with other cereals even in case of climate change with harsh temperature conditions. Moreover, it has greater ceiling temperatures for grain yield and is an underutilized crop with huge nutritional potential, which needs to be utilized fully (Krishnan and Meera, 2018). It is more resilient to extreme climatic events such as drought and water scarcity and can play a vital role in ensuring food and nutritional security in changing climatic scenarios, which is mounting to frightening proportions. Globally, it is the sixth most significant cereal crop after rice (*Oryza sativa*), wheat (*Triticum aestivum*), maize (*Zea mays*), barley (*Hordeum vulgare*) and sorghum (*Sorghum bicolor*). It is a staple food of 90 million poor people and extensively grown on 30-million-ha area in the arid and semi-arid tropical regions of Asia and Africa. It is also used for feed and fodder and accounts for almost half of the global millet production (Srivastava et al., 2020a). It is mainly cultivated on marginal lands under rainfed conditions and can sustain and produce a significant amount of grain even in drought-prone areas that receive an average annual precipitation of <250 mm (Nambiar et al., 2011). It surpasses all other cereals such as wheat, maize, rice, sorghum and barley because of its unique attributes like the C_4_ plant having high photosynthetic efficiency, more dry matter production capability, and survival under adverse agro-climatic conditions with lesser inputs and more economic returns (Nambiar et al., 2011). C_4_ plants have more ability to fix inorganic CO_2_ and more efficient in water utilization in comparison with C_3_ plants due to the presence of “Kranz” anatomy in leaves. Thus, being a C_4_ plant, pearl millet can account for 30% of global terrestrial carbon fixation along with other C_4_ plants such as maize and sorghum (Choudhary et al., 2020). It also possesses several advantages such as early maturity, drought tolerance, the requirement of minimal inputs, and usually free from biotic and abiotic stresses. Its inherent ability to endure high temperatures up to 42°C during the reproductive phase makes it suitable for growth in extremely hot summers under irrigations in northern Gujarat and eastern Uttar Pradesh of India, thus making it a climate-resilient crop.

It also possesses the huge capability to eliminate micronutrient deficiency among developing countries (Rai et al., 2012; Anuradha et al., 2017; Singhal et al., 2018) as it supplies 30–40% of inorganic nutrients and bestows affordable staple food along with ample amounts of iron and zinc (Rao et al., 2006). It has very high nutritional values and is a good source of energy, carbohydrates, crude fibers [resistant starch (RS), soluble and insoluble dietary fibers], soluble and insoluble fat, proteins (8–19%), ash, dietary fibers (1.2 g/100 g), antioxidants and fat (3–8%) with better fat digestibility, iron, and zinc in comparison with other major cereals (Uppal et al., 2015). It is also a rich source of vitamins such as riboflavin, niacin, and thiamine and minerals (2.3 mg/100 g) such as potassium, phosphorous, magnesium, iron, zinc, copper, and manganese (Weckwerth et al., 2020). It exhibits a better essential amino acid profile of protein in comparison with other cereals such as maize and rice. It contains lesser cross-linked prolamins leading to higher digestibility of the millet proteins. It has 74% polyunsaturated fatty acids (PUFAs) and rich in nutritionally sought-after omega-3 fatty acids such as oleic acid (25%), linoleic acid (45%), and linolenic acid (4%), which are considered best for health (Rooney, 1978; Nantanga, 2006; Dyall, 2015; Singh et al., 2018). It is a gluten-free grain that retains alkaline properties even after being cooked and is thus good for people suffering from gluten allergy. It owns a higher quantity of slowly digestible starch (SDS) and RS that account for lower glycemic index (GI) and is much preferred in recent times of transforming diets, food habits and the food industry (Satyavathi et al., 2020). It is a highly nutritious, non-acid-forming, non-glutinous food having several nutraceuticals and health beneficial properties along with high fiber content. It acts as a probiotic food for microflora present in our body and keeps us away from constipation. It is also capable of lowering cholesterol due to the presence of niacin in its grain. It contributes to an antioxidant activity with phytates, polyphenols etc. Consumption of various types of millets is considered to protect against certain types of cancer, cardiovascular diseases and various age-related diseases. Due to these useful properties, pearl millet is gaining a lot of popularity among health-conscious people all over the world. Due to its nutritional properties, pearl millet has been renamed as *nutri-cereal* (Gazette of India, No. 133 dated 13 April, 2018) and can play a vital role in overcoming malnutrition and ensure food and nutritional security.

In the present-changing climatic scenario, abiotic stresses entail a huge risk for plant growth and development leading to an over 50% decrease in the yield among the popular cereal crops (Bray et al., 2000). Almost 90% of the cultivable land is affected by various abiotic stresses globally, while only 10% of the agricultural land is free from these abiotic stresses (Dita et al., 2006). Drought and heat are the two most significant production constraints existing among the different environmental stresses. In this context, a crop species like pearl millet, which is resilient to higher temperatures and lower rainfall, can play a crucial role in fulfilling the increasing food demands of the growing population of the world. Pearl millet is mainly cultivated on marginal lands facing untimely and irregular rainfall patterns and environmental stresses due to its natural inbuilt capacity to survive in such areas and withstand abiotic stresses such as drought, salinity, heat etc. (Serba and Yadav, 2016). Genetic improvement of pearl millet through increased production was realized using hybrid technology and conventional breeding methods of selection (Yadav and Rai, 2013; Yadav et al., 2021) but later, various biotechnological and genomic approaches were used for further improvement (Varshney et al., 2017; Bollam et al., 2018; Ambawat et al., 2020). Genetic maps, next-generation sequencing (NGS), genotyping-by-sequencing (GBS), genome-wide association studies (GWAS), synteny studies, expression profiling, fine QTL mapping, candidate gene identification and genetic engineering, gene pyramiding, bioinformatics and systems biology are some of the useful platforms, which are further being used for the genetic improvement of this nutricereal. Recently reported genome sequence information in the year 2017 can speed up gene innovation and trait mapping and can help in the understanding of several complicated gene pathways and their interactions (Varshney et al., 2017). Further, the challenge remains to characterize thousands of genes crucial for abiotic stress response and tolerance. Similarly, there is a high need to identify the lines that can use nitrogen efficiently as most of the agro-ecologies where pearl millet is grown have low N in the root zone soil strata.

In addition, integrated knowledge on genomics as well as transcriptomics, proteomics and metabolomics is also beneficial for advancements and biofortification of pearl millet (Dita et al., 2006; Lata, 2015; Anuradha et al., 2017; Ambawat et al., 2020). Hence, there is a need to focus on this very important crop and harness its suitability to adverse conditions and utilize its inbuilt capacity to ensure global food and nutritional security. Here, we have reviewed the importance of pearl millet in the present-changing climatic scenario for food and nutritional security and various advances made in the pearl millet improvement program.

## Status of Pearl Millet Production

**Figure 1:**
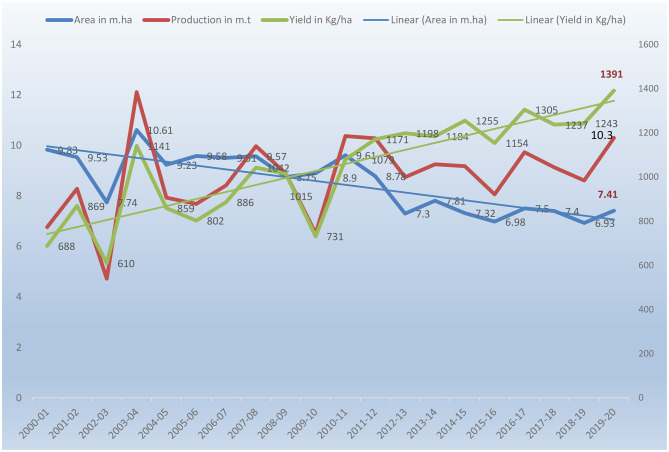
Area, production and productivity of Pearl millet in India since 2000.

Pearl millet is a descendent of the wild West African grass and was domesticated over 4,000 years ago in the West African Sahel, spreading later to East Africa and India (Sharma et al., 2021). Now it is being cultivated over 30 million ha worldwide, with the majority of the crop grown in Africa (>18 million ha) and Asia (>10 million ha) (Raheem et al., 2021). Around 90 million people in the Sahelian region of Africa and northwestern India consume pearl millet grain as a staple food (Srivastava et al., 2020b). Jukanti et al. (2016) have reviewed the origin and evolutionary history of pearl millet. It is the sixth major cereal crop in the world followed by maize, rice, wheat, barley and sorghum and cultivated on 30 million ha in the arid and semi-arid tropical regions of Asia and Africa accounting for around half of the global millet production with 60% of the cultivation area in Africa, followed by 35% in Asian countries. In terms of area and production, India is the largest producer of pearl millet. During 2010–2012, the average pearl millet area in India was 8.5 million ha and the average production was 9.4 million tons. It is taken up in an area of 6.93 million ha with an average production of 8.61 million tones and 1,243 kg/ha productivity (Directorate of Millets Development, 2020). The trends of area, production, and productivity over the years are shown in Figure 1. In Africa, the west/central Africa (WCA) region (Nigeria, Niger, Chad, Mali, and Senegal), and east/southern Africa (ESA), which includes Sudan, Ethiopia, and Tanzania, are the two main areas of pearl millet cultivation. Pearl millet is the third major crop in sub-Saharan Africa with Nigeria, Senegal, Chad, Mali, Niger and Burkina Faso as the major producing countries and has socio-economic, food/feed, health and environmental impact on the resource-poor people of Africa. WCA is the largest pearl millet-producing region in Africa and the world, accounting for 95 % of the total area in WCA (Jukanti et al., 2016). West Africa is the largest producer led by Nigeria (41%), Niger (16%), Burkina Faso (7%), Mali (6.4%), and Senegal and Sudan (4.8%). In Africa, it is produced in 18.50 million ha by 28 countries with a yield of 11.36 million tons covering 30% different areas of the continent in diverse agro-ecologies. It is 49% of the global millet area with great significance (FAO, 2019).

It is extensively cultivated in India and is the fourth most extensively grown cereal crop after rice, wheat and maize. Rajasthan, Maharashtra, Uttar Pradesh, Gujarat, and Haryana are the major pearl millet-growing states, contributing 90% of the total production in India. Out of this, Rajasthan contributes a maximum of around 4.283 million 5 tonnes, followed by Uttar Pradesh (1.302), Haryana (1.079), Gujarat (0.961), Maharashtra (0.66), and Tamil Nadu (0.084). It is mainly cultivated in the rainy (*kharif* ) season (June/July-September/October) but it is also grown in some parts of Gujarat, Rajasthan, and Uttar Pradesh during the summer season (February-May), while it is also cultivated in states of Maharashtra and Gujarat at a small scale during the post-rainy (*rabi*) season (November-February). As millets are climate-smart crops with nutritional value, they are rightly termed as *nutricereals* (Gazette of India, No. 133 dated 13 April, 2018). In addition, to include millets into the mainstream and exploit its nutritionally superior qualities and promote its cultivation, Government of India has declared Year 2018 as the “Year of Millets” and FAO Committee on Agriculture (COAG) forum has declared Year 2023 as “International Year of Millets.”

## Pearl Millet Improvement

Indian Council of Agricultural Research started pearl millet breeding in India in the 1940s, and X1 and X2 were the two chance hybrids released in India for commercial utilization in the fifties (Yadav and Rai, 2013). Pearl millet improvement programs were implemented in several phases. During phase I, breeders mainly focused on the flowering habit, mode of pollination, germplasm evaluation and enhancement, genetics and cytogenetics of agronomically important traits, cytoplasmic male sterility (CMS) etc. Thus, initially, efforts were put forward towards the identification and use of dwarfing genes for enhancing the yield using locally adapted materials and various Open Pollinated Varieties (OPVs) were developed. As a result of this, pearl millet hybrid research has gained importance in India and the productivity was 4.5 kg/ha/year during this phase (Yadav et al., 2019). By the 1960s, hybrid development became the major objective of breeding for enhancing pearl millet production and productivity. Hybrid “HB-1” (Hybrid Bajra-1) was the first pearl millet hybrid released in 1965 (Athwal, 1965) followed by a series of hybrids between 1965 and 1988 and during phase II, an annual increase of 6.6 kg/ha was achieved in productivity.

The genetic improvement program progressed effectively initiating from the selection of local and traditional germplasm to the development of high-yielding hybrids possessing inbuilt tolerance and resistance to climatic stresses such as drought and heat along with different diseases. These hybrids were grown on 70% of the total pearl millet area, resulting in a 124% enhancement in productivity since 1986–1990. ICAR-All India Coordinated Research Project on Pearl Millet has developed several precise production and protection technologies for different agro-ecological regions of different states since its beginning in 1965. Till now, a total of 180 hybrids and 62 varieties have been identified and released for growing in different agro-ecological regions of India through ICAR-All India Coordinated Research Project on Pearl millet (Satyavathi et al., 2020). Several genetically diverse CMS lines have been developed and used along with marker-assisted breeding (MAB) and marker-assisted backcrossing, resulting in an increased productivity to 19.0 kg/ha/year during phase III (Yadav et al., 2019).

**Figure 2:**
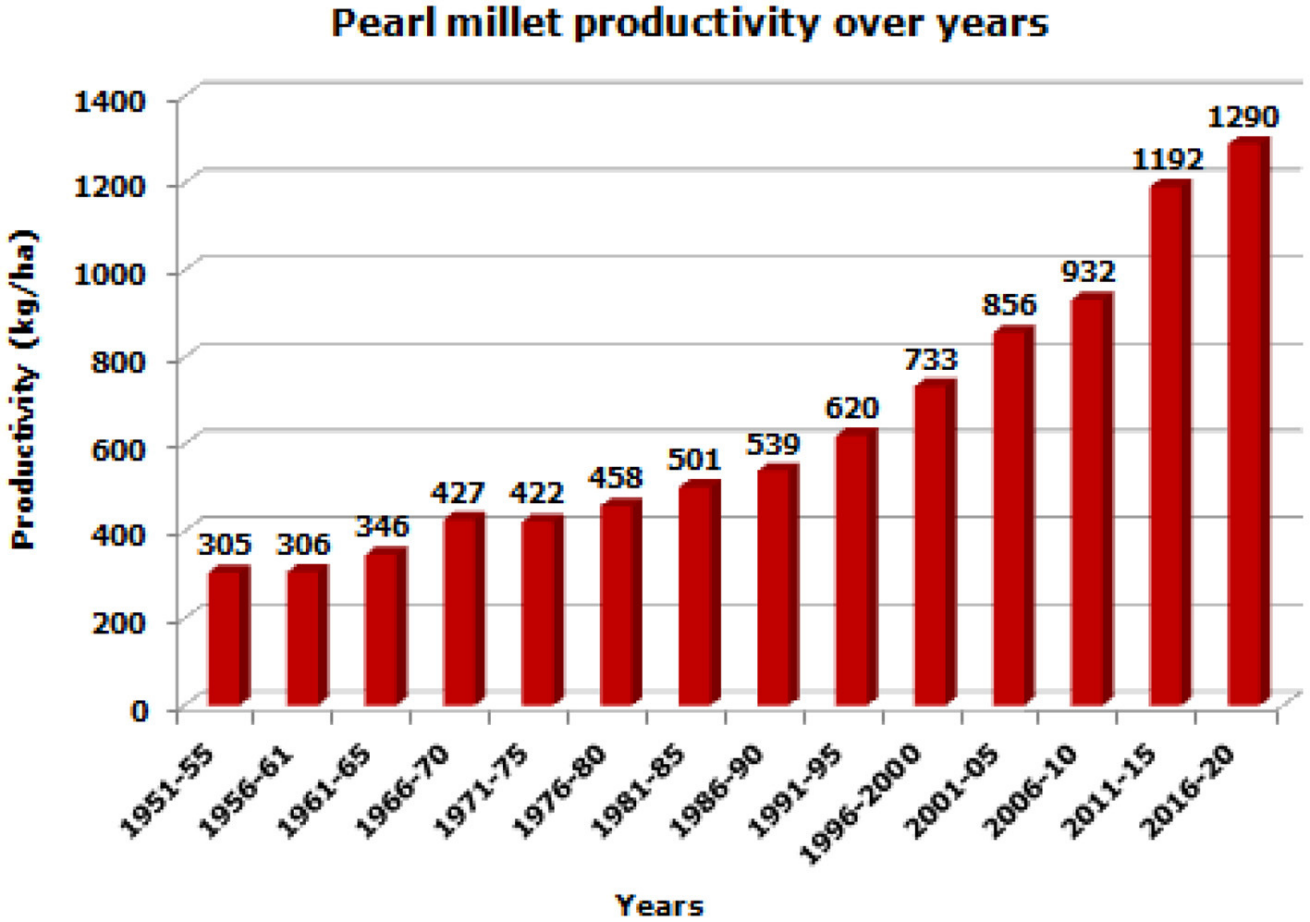
Trends in pearl millet productivity over years (based on 5-year average).

During the fourth phase, great emphasis was laid on the genetic diversity of seed and pollinator parents and adaptation to niche areas, resulting in the release of a large number of cultivars and a significant increase in productivity 31.1 kg/ha/year, which was almost five times in comparison with Green Revolution Era (Govindaraj et al., 2010; Kumara et al., 2014; Yadav et al., 2019). In the next phase, biofortification of the grain for micronutrients, largely for zinc and iron and application of molecular techniques were focused to speed up the cultivar development program (Rai et al., 2013; Kanatti et al., 2016, Kumar et al., 2016, 2018; Anuradha et al., 2017; Singhal et al., 2018; Govindaraj et al., 2019). These cultivars were widely adopted by Indian farmers resulting in enhanced crop productivity from 305 kg/ha during 1951–1955 to 998 kg/ha during 2008–2012 and 1,243 kg/ha during 2018–19 (Yadav and Rai, 2013; Satyavathi et al., 2020) (Figure 2). The productivity during the 5 years starting from 1951 to 1955 (305 kg/ha) has increased to 1,290 kg/ha for the 5 years 2016–2020. The productivity improvement is four-fold, or 400%. Compared to the previous 5 years term of 2011–2015, the productivity was 1,192 kg/ha, while it is 1,290 kg/ha for the 5 years 2016–2020. The improvement is 7.6% compared to the period of 2011–2015. This improvement is due to the combined contribution of the development of high-yielding hybrids, varieties, biofortified genotypes, improved production practices, technologies, and recommendations coupled with adoption by farmers.

Several efforts were taken for crop improvement in pearl millet using conventional as well as advanced molecular and genomic tools as listed in Tables 1, 2. Pearl millet is the first crop in which marker-assisted-selection (MAS) strategies and tools were applied to get improved varieties. Yadav et al. (2021) discussed various past strategies and future approaches to accelerate genetic gains to meet future demand. Further, they also emphasized the importance of genome editing, pre-breeding, precision phenotyping protocols and speed breeding approaches for pearl millet improvement and enhanced genetic gains. On the other hand, due to a lack of knowledge and non-availability of ideal dose of fertilizer among poor farmers, it becomes difficult to harvest the real yield potential. Hence, the yield improvement of pearl millet under low nitrogen input is indeed beneficial for economic and environmentally sustainable cultivation. Pujarula et al. (2021) studied the genetic variation for nitrogen-use efficiency (NUE) among a set of 380 diverse pearl millet lines. Thus, such studies of different physiological traits and their relationship with grain yield are very important for understanding the complex nature of NUE.

**Table 1:** Achievements and milestones in pearl millet improvement through conventional/heterosis breeding.

**Year**	**Achievements**	**References**
1940	Indian Council of Agricultural Research started Pearl millet breeding in India.	Yadav and Rai, 2013; Singh et al., 2014
1950	X1 and X2 were the two chance hybrids released in India for commercial utilization.	Yadav and Rai, 2013; Singh et al., 2014
1965	First pearl millet hybrid, Hybrid “HB-1” (Hybrid Bajra-1) was released.	Athwal, 1965
1965	Establishment of the ICAR-All India Coordinated Research Project on Pearl Millet	Yadav and Rai, 2013
1997-2008	Effective phenotypic screening techniques and resources were developed, and resistance breeding programs were developed.	Thakur and King, 1988; Singh et al., 1997; Hash et al., 1999; Hash and Witcombe, 2002; Jones et al., 2002; Thakur et al., 2008
1974–2020	Identification and release of 175 hybrids and 62 varieties for cultivation in different agro-ecological zones of India	Satyavathi et al., 2020
2018	Identified heterotic groups for grain yield and a total of 343 hybrid parental [maintainer (B-) and restorer (R-)] lines were genotyped with 88 polymorphic SSR markers.	Ramya et al., 2018
2021	Genetic variation studies for NUE among a set of 380 diverse pearl millet lines	Pujarula et al., 2021
2021	Studied expression of heat stress-responsive gene Pghsp	Sankar et al., 2021

**Table 2:** Achievements and milestones in pearl millet improvement through molecular and advanced genomic tools.

**Year**	**Achievements**	**References**
**Molecular markers**		
1994	Study of 200 samples of varied pearl millet lines using RFLP markers to reveal polymorphism	Liu et al., 1994
1995	Development of amplified fragment length polymorphism (AFLP) markers for pearl millet using nuclear genomic sequences	Devos et al., 1995
2000	163 AFLP markers were used to study genetic variability within and between pearl millet landraces	Busso et al., 2000
2001	Development of STSs markers from BAC clones	Allouis et al., 2001; Qi et al., 2001
2002	Genetic diversity was studied within and between 504 landraces of core collection using RFLP probes	Bhattacharjee et al., 2002
2003	18 SSR markers were developed from genomic sequences in pearl millet	Budak et al., 2003
2004	A consensus map of 353 RFLP and 65 SSR markers was developed for the first time.	Qi et al., 2004
2005	SSCP-SNP primes were developed from pearl millet EST collections	Bertin et al., 2005
2008	EST-based SSRs were developed in pearl millet	Senthilvel et al., 2008
2011	DArT platform was established for pearl millet, and 574 polymorphic DArT markers were mapped and used to genotype a set of 24 diverse pearl millet inbred lines	Supriya et al., 2011
2012	Conserved intron-specific primers (CISP) were developed from EST sequences using parents of two mapping populations for 18 genes	Sehgal et al., 2012
2013	Consensus linkage maps based on SSRs were constructed using four RIL populations	Rajaram et al., 2013
2013	Genetic diversity was analyzed in a novel set of restorer lines using SSR markers in pearl millet	Satyavathi et al., 2013
2014	Development of ISSR-based SCAR markers in pearl millet	Jogaiah et al., 2014
2015	Identification of single nucleotide polymorphisms (SNPs) using GBS platform	Hu et al., 2015; Sehgal et al., 2015
2020	A panel of 21,663 SNP markers was developed	Diack et al., 2020
2020	Morphological and molecular genetic diversity analysis of pearl millet (*Pennisetum glaucum*) maintainers and restorers.	Chandra et al., 2020
**QTL mapping**		
1995–2007	DNA markers have been established for around 60 different putative DM resistance QTLs in pearl millet	Jones et al., 1995, 2002; Hash et al., 1999; Breese et al., 2002; Hash and Witcombe, 2002; Gulia et al., 2007
1998	Identified molecular markers for three rust loci and one *Magnaporthe* resistance locus in pearl millet.	Morgan et al., 1998
1999, 2002	QTL was identified for grain and stover yield in pearl millet under terminal drought stress conditions	Yadav et al., 1999, 2002
2005	Evaluated putative DT-QTL on LG2	Bidinger et al., 2005; Serraj et al., 2005
2007	Three major QTLs were identified on LG 2, LG 3, and LG 4 for grain yield under variable post-flowering water conditions	Bidinger et al., 2007
2010	Identified QTL responsible for terminal drought tolerance	Kholová et al., 2010
2011	Identified one major QTL on LG2 for grain yield and drought tolerance	Yadav et al., 2011
2012	Co-mapped alleles to terminal drought tolerance QTL	Kholová et al., 2012
	Investigated the effects of DT-QTL on LG 2 under salinity stress	Sharma et al., 2014
2015	Detected four QTLs linked to high transpiration rate	Aparna et al., 2015
2016	A major QTL for rust resistance was identified on LG 1 using the cross 81B-P6 × ICMP 451-P8	Ambawat et al., 2016
2018	QTLs were identified on LG 1 and LG 4 for downy mildew (DM) resistance in pearl millet	Taunk et al., 2018
2018	Identified five QTLs for early drought stress conditions and stay-green trait	Debieu et al., 2018
2018	Role of chitosan nanoparticles was explored for resistance against pearl millet downy mildew	Siddaiah et al., 2018
2019	Introgressed DT-QTLs into hybrid HHB 226 from 863 B, the male parent HBL 11	Jangra et al., 2019
2019	Five QTLs were identified for resistance to three different pathotype isolates of *S. graminicola*	Chelpuri et al., 2019
**Advanced genomic tools**		
2006	Improved HHB67 was developed using the marker-assisted selection	Hash et al., 2006
2009	GWAS approach was used to dissect complex traits in pearl millet	Saïdou et al., 2009
2015	SNP markers were identified using genotyping-by-sequencing (GBS) pearl millet and high-density maps were constructed.	Hu et al., 2015; Moumouni et al., 2015
2015	PMiGAP was established and used for fine mapping of a drought tolerance DT-QTL on LG2 using candidate gene-based association mapping (AM) approach.	Sehgal et al., 2015
2016	Transcriptomic analysis was done using NGS tool to understand the mechanisms underneath resistance to downy mildew in pearl millet	Kulkarni et al., 2016
2016	16,650 SNPs and 333,567 sequence tags across all seven chromosomes have been identified for leaf spot resistance using GBS platform.	Punnauri et al., 2016
2017	A draft genome sequence of *S. graminicola pathotype* 1 of 299,901, 251 bp in length, N_50_ of 17,909 bp with a minimum of 1 Kb scaffold size was assembled	Nayaka et al., 2017
2017	Whole-genome sequence of pearl millet was deciphered	Varshney et al., 2017
2018	A total of 392,493 SNPs identified using GWAS on a panel of 188 inbred lines	Debieu et al., 2018
2018	Sequencing data were generated for RAD-seq and tGBS using genomic selection (GS) schemes in pearl millet.	Liang et al., 2018
2018	Transcriptome analysis for drought stress response using RNA-Seq approach in pearl millet	Dudhate et al., 2018; Jaiswal et al., 2018
2019	Genome of *Magnaporthe grisea* strain PMg_Dl and was sequenced, and 13.1-Gb PE reads were generated	Prakash et al., 2019
2019	Studied the genetic diversity, population structure, and linkage disequilibrium in 398 accessions using GBS	Serba et al., 2019
2019	Genetic diversity of 130 forage-type hybrid parents of pearl millet was investigated using GBS-derived 7870 SNPs	Ponnaiah et al., 2019
2020	Characterized 309 inbred lines by 54,770 GBS-SNPs and reported higher nucleotide diversity in the panel derived from landraces and improved varieties from Africa and India.	Kanfany et al., 2020
2020	Comparative transcriptomics at vegetative and flowering stage was done using RNAseq analysis in a drought-tolerant (PRLT2/89-33) genotype to discover underlying genes to drought tolerance	Shivhare et al., 2020
2020	6920 genes and 6484 genes differentially expressed under heat stress and drought stress were identified using RAD-GBS	Sun et al., 2020
2020	Heterotic groups were defined and 0.9 million SNPs clustered into 12 R- and 7 B-line groups	Gupta et al., 2020
2021	Importance of wild relatives of pearl millet germplasm was harnessed for germplasm enhancement and improving biotic stress tolerance in pearl millet	Sharma et al., 2021
**Quality improvement**		
2009–2020	Implementation of biofortification approaches in pearl millet for high Fe and Zn	Govindaraj et al., 2009, 2019, 2020; Kanatti et al., 2014; Rai et al., 2016; Anuradha et al., 2017
2016, 2017	Reported diversity in the rancidity profile of pearl millet genotypes	Datta Mazumdar et al., 2016; Goyal and Chugh, 2017
2016	Identified two QTLs for grain Fe content on LG3 and LG5, and two QTLs for grain Zn content on LG3 and LG7 using replicated samples of 106 pearl millet RILs (F_6_) derived from ICMB 841-P3 × 863B-P2 cross.	Kumar et al., 2016
2017	Identified favorable alleles for grain iron and zinc content through AM in pearl millet.	Anuradha et al., 2017
2018	Reported large effect of Fe and Zn content QTLs using DArT and SSR markers in ICMS 8511-S1-17-2-1-1-B-P03 × AIMP 92901-S1-183-2-2-B-08 cross.	Kumar et al., 2018
2019	RAD-GBS was used and three GS models were implemented and compared using grain yield and dense molecular marker information of pearl millet	Jarquin et al., 2019
2020	Developed a rancidity matrix (RM) and classified pearl millet genotypes into low, medium, and high rancid groups	Goswami et al., 2020; Kumar et al., 2020
2020	Identified candidate genes for grain Fe and Zn contents in pearl millet	Mahendrakar et al., 2020
2021	Mapped multienvironment QTLs for grain iron and zinc contents in pearl millet.	Singhal et al., 2021

## Climate Resilience in Pearl Millet and Genomic Resources

Pearl millet can survive and produce a large quantity of grain, whereas other cereals such as rice, wheat, maize, sorghum and barley may fail to provide economic benefits under adverse conditions and poor soil. It can provide multiple securities in form of food, fodder, livelihood, nutrition health and ecological benefits, whereas wheat and rice provide only food security, thus making it a crop of agricultural security. Its ability to withstand higher temperature and survival in drought-prone areas and cultivation in parts of Gujarat and eastern Uttar Pradesh of India during hot summers makes it a climate-resilient crop for overcoming the adverse effects of the changing climate (Gupta et al., 2015). National Agricultural Research System (NARS) in India and International Crops Research Institute for the Semi-Arid Tropics (ICRISAT) have played a significant role in developing various improved breeding and parental lines of prospective hybrids. A total of 21,392 germplasm accessions, including 750 accessions of wild species of genera *Pennisetum* and *Cenchrus*, collected from 50 countries are conserved at the ICRISAT Genbank, while 8,284 accessions are conserved at the National Bureau of Plant Genetic Resources (NBPGR), New Delhi, India. These lines have been widely used in breeding programs in both the public and private sectors for the development and commercialization of a large number of hybrids (public 70 and private sectors 105 were under cultivation in 2019).

Various molecular markers and genomic tools have been developed and applied for QTLs/genes identification, genetic diversity, and MAB to enhance pearl millet breeding by exploring its genetic potential at the molecular level (Serba and Yadav, 2016; Anuradha et al., 2017; Bollam et al., 2018; Kumar et al., 2018; Singhal et al., 2018; Ambawat et al., 2020; Srivastava et al., 2020a,b). Various molecular markers developed for pearl millet include restriction fragment length polymorphism [RFLP (Liu et al., 1994)], random amplified polymorphic DNA (RAPD), amplified fragment length polymorphism [AFLP (Devos et al., 1995)], sequence-tagged sites [STSs (Allouis et al., 2001)], simple sequence repeat [SSRs (Qi et al., 2004; Meena et al., 2020; Srivastava et al., 2020a)], single-stranded conformation polymorphism-SNP [SSCP-SNP (Bertin et al., 2005)], expressed sequence tag-derived simple sequence repeats [EST-SSRs (Senthilvel et al., 2008; Rajaram et al., 2013)], DArT array technology [DArTs (Senthilvel et al., 2010; Supriya et al., 2011)], conserved intron-specific primers [CISP (Sehgal et al., 2012)], and single nucleotide polymorphisms [SNPs (Sehgal et al., 2012)]. High allelic variation and polymorphism were recorded between pairs of parental lines of pearl millet after screening with SNP markers, and they were also mapped on all the seven linkage groups reflecting the distribution of the markers in the genome (Sehgal et al., 2012). Similarly, a panel 21,663 SNP markers were also discovered, depicting > 5% of minor allele frequencies by Diack et al. (2020). Different types of molecular markers and genomic approaches ultimately provide a systematic knowledge on plant biology enabling MAB, which can accelerate the process of the development of new and resistant hybrids/varieties.

MAS and gene introgression into desirable genetic background have been proved to be very efficient for crops improvement as it reduces the cumbersome procedure of phenotypic evaluation and selection. Pearl millet is one of the crops where MAB approach has been applied to develop downy mildew-resistant variety “Improved HHB 67” (Hash et al., 2006). Later, studies were also conducted to identify and map major QTLs affecting abiotic stress tolerance in pearl millet (Yadav et al., 1999, 2002, 2004, 2011; Serraj et al., 2005; Bidinger et al., 2007; Kholová et al., 2012, 2013; Tharanya et al., 2018). The first QTL mapping study was conducted on drought tolerance using recombinant inbred line (RIL) mapping population from the cross H 77/833-2 × PRLT 2/89-33, and a major QTL for drought tolerance (DT-QTL) was mapped on LG 2 explaining 32% variation for grain yield (Yadav et al., 1999, 2002). Subsequently, fine mapping of the DT-QTL was done and several markers mapped on this region of LG2 were identified, which were found to be linked with traits of drought tolerance, including delayed leaf senescence and leaf rolling under drought stress (Sehgal et al., 2012). Subsequently, Sharma et al. (2014) investigated the effects of DT-QTL under salt stress conditions. Later, Sehgal et al. (2015) utilized PMiGAP for the first time for fine mapping of a drought tolerance (DT) QTL (localized on linkage group 2) using candidate gene-based AM approach. This PMiGAP can be used as a genetic resource for GWAS studies in pearl millet.

Recently, NGS and other advanced high-throughput assays were used to sequence the pearl millet genome, which will prove useful for its improvement and enhancing different yield and yield-related traits along with major biotic and abiotic stress tolerance and nutritionally significant traits worldwide (Varshney et al., 2017). Consequently, GBS, which allows simultaneous SNP discovery and genotyping, has been also used extensively in pearl millet to characterize germplasm (Poland and Rife, 2012; Ramu et al., 2013; Sehgal et al., 2015; Ponnaiah et al., 2019). GBS was used to construct a high-density genetic map with a more uniform distribution of markers in pearl millet from a biparental population in comparison with maps constructed earlier (Moumouni et al., 2015). Similarly, 83,875 SNP markers were identified from *Pst*I-*Msp*I reduced representative libraries of pearl millet by GBS, revealing a wide genetic variation in germplasm collection (Hu et al., 2015). A total of 398 accessions were used to study the genetic diversity, population structure, and linkage disequilibrium using GBS (Serba et al., 2019). Recently, Kanfany et al. (2020) characterized 309 inbred lines derived from landraces and improved varieties of India and Africa by 54,770 GBS-SNPs and reported higher nucleotide diversity in the panel.

Various genetic and genomic resources like GWAS and genomic selection (GS) have been also established in pearl millet, which is useful in trait discovery and whole-genome scan studies. Phenotypic data are combined with GBS data, and different genomic regions governing traits of interest are identified using GWAS. It can be used to exploit the natural diversity available in population or germplasm panels and can be used to increase mapping resolution in comparison with linkage mapping populations (Srivastava et al., 2020a). GS is eventually the extension of marker-assisted selection, and using it, genome-wide molecular markers are targeted and promising individuals are selected. It calculates the breeding values by combining marker genotypic data with phenotypic data collected from several environments and pedigree or kinship for improvement in quantitative traits and can ultimately speed up the genetic gain (Meuwissen et al., 2001; Goddard and Hayes, 2007; Heffner et al., 2009). Srivastava et al. (2020a) reviewed various case studies and the development of various whole-genome prediction/GS models based on GWAS which will be highly useful to explore the underlying genetics associated with pearl millet. The use of GWAS approach to dissect complex traits was initially reported in pearl millet by Saïdou et al. (2009). In this study, they revealed the genetic factors controlling the variations in flowering time at the phytochrome C (PHYC) (866 bp) locus, which plays a major role in crop adaptation mechanism. Later, Saïdou et al. (2014) investigated an extra region of 100 Kb around the *PHYC* gene using the same panel of 90 inbred lines to identify tightly linked best candidate markers. Debieu et al. (2018) also performed GWAS on a panel of 188 inbred lines of West Africa to identify QTLs associated with stay-green trait and biomass production in early drought stress conditions. In addition, different “omics” technologies such as transcriptomics, proteomics, and metabolomics can be also useful for quantitative and qualitative analyses of gene expression allowing more precise use of MAS and transgenic technologies. RNA sequencing (RNA-Seq) is widely used among the different transcriptome analysis methods as it can efficiently detect unknown genes and novel transcripts and has much potential to study gene expression and their regulating pathways (Hrdlickova et al., 2017). The transcriptome analysis of pearl millet can reflect new prospects into gene regulatory networks existing in this crop under abiotic stress conditions. Stress-regulated pathways in pearl millet can be studied by the detection and characterization of stress-responsive genes *via* transcriptomics and then different approaches can be followed for improving stress tolerance/resistance in millet (Mishra et al., 2007). Several transcriptomic studies in pearl millet have been used to reveal the functions of some salinity stress-responsive genes such as PgDHN, PgDREB2A, PgVDAC, PgNHX1 (Desai et al., 2006; Mishra et al., 2007; Verma et al., 2007; Agarwal et al., 2010; Reddy et al., 2012; Singh et al., 2015). Recently, complete transcriptome analysis has been done in pearl millet for drought stress response (Dudhate et al., 2018; Jaiswal et al., 2018; Shivhare et al., 2020). Dudhate et al. (2018) unraveled the molecular mechanism governing drought tolerance in two pearl millet inbred lines, ICMB 843 and ICMB 863, using RNA-Seq approach, and it is the first report of the study of drought tolerance by RNA sequencing in pearl millet. More recently, an important study on RNAseq analysis was performed to assess the comparative transcriptomics at the vegetative and flowering stage in a drought-tolerant (PRLT2/89-33) genotype to discover underlying genes to drought tolerance (Shivhare et al., 2020).

Proteomics is another important technology to get information on protein concentrations, post-translational modifications (PTMs), protein–protein interaction, structures linked with stress tolerance, regulatory functions of proteins encoded by genes (Ghatak et al., 2017). Identification and characterization of stress-responsive genes and their proteins from pearl millet can help in defining stress-regulated pathways. Further, it can help design strategies to improve stress tolerance/resistance of pearl millet as well as other related crop plants. Several proteomic studies have been also carried out in pearl millet, which can provide a framework to investigate C_4_ photosynthesis in pearl millet in more depth (Ghatak et al., 2016, 2021; Weckwerth et al., 2020). A shotgun proteomics approach (GEL-LC-Orbitrap-MS) was used by Ghatak et al. (2016) to identify 2,281 proteins from different tissues of pearl millet (root, seed, and leaf) showing significant changes under drought stress condition. Thus, a lot of information has been generated for the improvement of this crop but still, there is a big challenge to identify various crucial genes responsible for its adaption to survival under different abiotic stresses conditions. In addition, the identification of genomic regions governing NUE and its use in pearl millet breeding programs *via* MAS can be also exploited for survival under adverse conditions. Moreover, exploring several new and advanced genomic tools will be also beneficial for the advancement of this crop to harness its suitability to adverse conditions and utilize its inbuilt capacity to ensure global food and nutritional security.

## Nutritional Security

**Figure 3:**
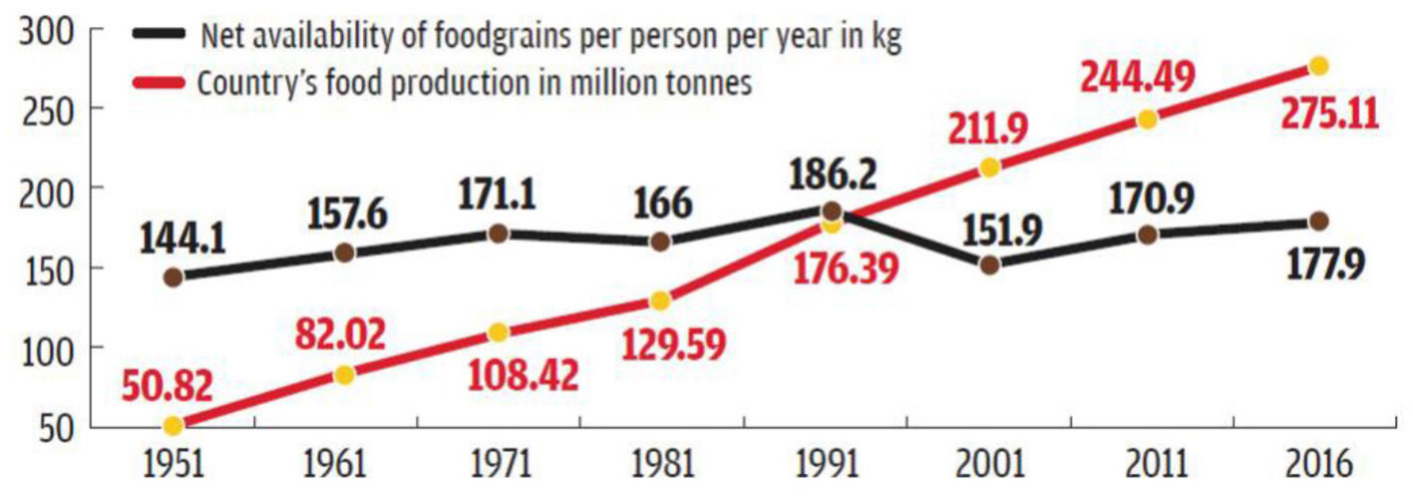
Trends in food grain production and per capita availability in India. Adapted from: Agriculture Statistic, Ministry of Agriculture and Family Welfare.

Food grain production has increased but still, to feed the growing population and meeting out good health of the people in the present situation, nutritional security is very important (Figure 3).

Intake of all essential macronutrients and micronutrients (vitamins and minerals) through a balanced and diversified diet in sufficient quantities is very vital for an active and healthy life. Nutritional insecurity is a major challenge for the world's growing population, which mainly depends on a micronutrient-deficient cereal-based diet. Pearl millet is the primary source of energy for the semi-arid tropics and drought-prone regions of Asia and Africa after the major cereals such as wheat, rice, maize and sorghum due to its nutritionally superior grain enriched with high amounts of essential amino acids, proteins, better fat digestibility, vitamins and minerals (Table 3). It is a good source of carbohydrate, energy, RS, 92.5% dry matter, fat (5–7%), ash (2.1%), dietary fiber (1.2/100 g), 13.6% crude protein, quality protein (8–19%), 63.2% starch, α-amylase activity, minerals (2.3 mg/100 g), vitamins A and B, and antioxidants such as coumaric acids and ferulic acid (Goswami et al., 2020). In addition, it is rich in unsaturated fatty acids (75%) and phytic acid, which are considered to be useful in lowering cholesterol and phytate, which in turn reduces cancer risk. It also exhibits antioxidant activities due to the presence of polyphenols, anthocyanins, phytates, phytosterols, tannins and pinacosanols and thus plays a significant role in aging. It is also enriched with many essential amino acids except lysine and threonine and has relatively higher methionine. Being gluten-free, it is extremely useful for people suffering from celiac diseases who are generally allergic to the gluten content of wheat and other cereals. Pearl millet is exceptionally useful for people suffering from diseases like diabetes, obesity, diabetic heart disease, atherosclerosis and metabolic diseases due to its health beneficial properties (Kumar et al., 2020).

**Table 3:** Nutritional comparison of pearl millet with sorghum, rice, and wheat (in 100 g grain).

**Contents**	**Crop**			
	**Pearl millet**	**Sorghum**	**Rice**	**Wheat**
Carbohydrates (g)	61.8	67.7	78.2	64.7
Protein (g)	10.9	09.9	07.9	10.6
Fat (g)	5.43	1.73	0.52	1.47
Energy (Kcal)	347	334	356	321
Dietary fiber (g)	11.5	10.2	02.8	11.2
Calcium (mg)	27.4	27.6	07.5	39.4
Phosphorus (mg)	289	274	96	315
Magnesium (mg)	124	133	19	125
Zinc (mg)	2.7	1.9	1.2	2.8
Fe (mg)	6.4	3.9	0.6	3.9
Thiamine (mg)	0.25	0.35	0.05	0.46
Riboflavin (mg)	0.20	0.14	0.05	0.15
Niacin (mg)	0.9	2.1	1.7	2.7
Folic acid (μg)	36.1	39.4	9.32	30.1

*Adapted from: NIN, Hyderabad, 2018*.

It is also called the “*Powerhouse of Nutrition*” due to its richness with essential nutrients in good quantity and quality, which are vital for leading healthy and nutritious life. Pearl millet has elevated contents of various macronutrients as well as micronutrients like iron, zinc, magnesium, calcium, phosphorous, copper, manganese, riboflavin, and folic acid. Owing to such excellent nutritional values, it is gaining popularity and is preferred by people all over the world including developed countries.

**Figure 4:**
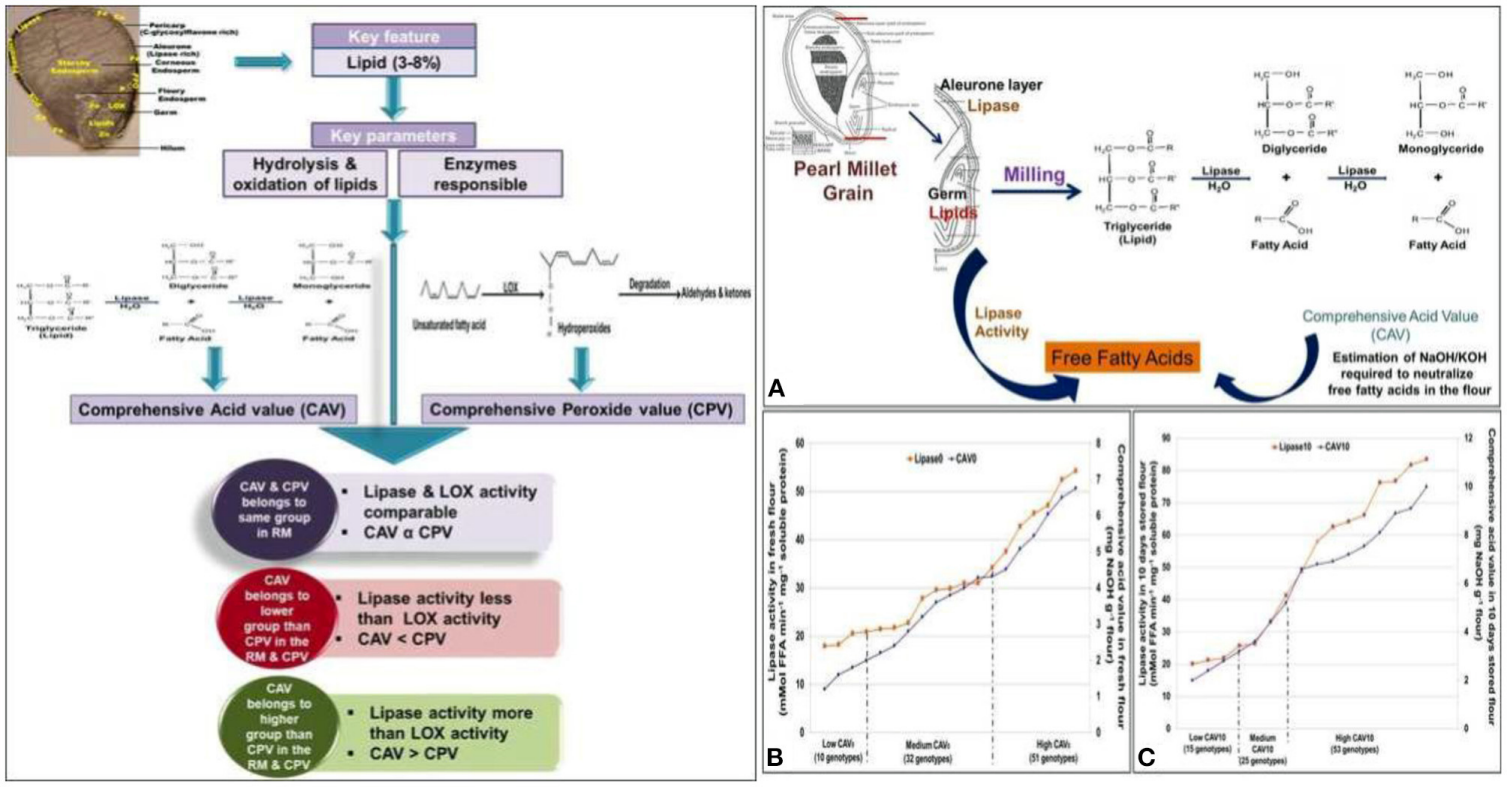
Mechanism of action and role of lipase activity for shelf life in pearl millet flour (Adapted from: Goswami et al., 2020). **(A)** Correlation between lipase activity and comprehensive acid value (CAV). **(B)** Correlating the lipase activity and CAV of pearl millet genotypes in group I (freshly milled flour). **(C)** Correlating the lipase activity and CAV of pearl millet genotypes in group II (10-day stored flour).

Despite its nutritional superiority, the consumption of pearl millet flour is restricted to very few specific regions of the world because of the poor shelf life of the flour and the development of rancidity or off-odor on storage (Rani et al., 2018). Rancidity is caused by oxidative/hydrolytic enzymes such as lipase, lipoxygenase (LOX), etc., where they hydrolyze the triacylglycerol (TAG) to diacylglycerols, glycerol, monoglycerol, and free fatty acids (Manley and Mayer, 2012) (Figure 4).

**Figure 5:**
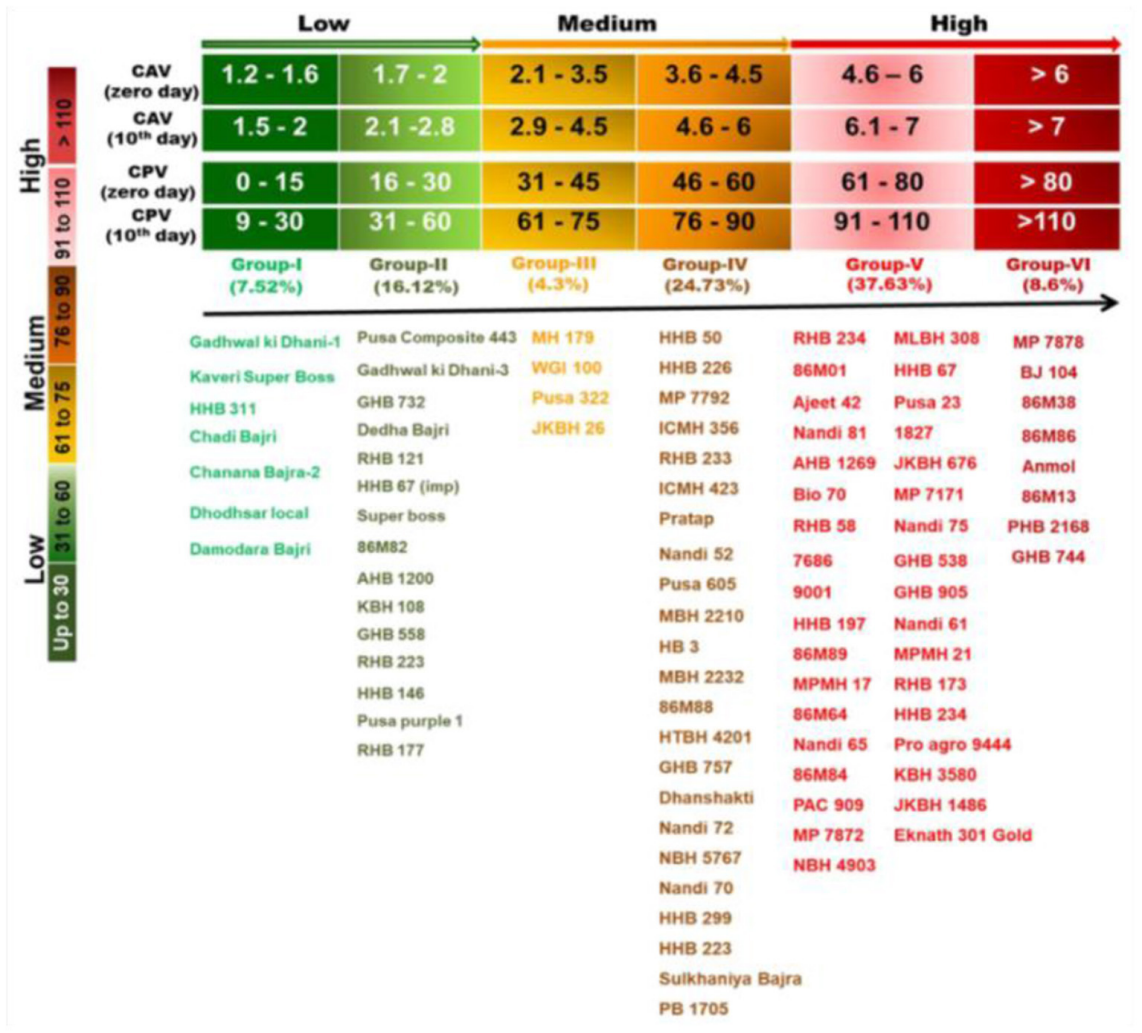
Rancidity matrix to measure rancidity in the pearl millet flour (Adapted from: Goswami et al., 2020).

Goswami et al. (2020) categorized 93 diverse genotypes of pearl millet into low, medium and high rancid groups. They developed and validated a rancidity matrix (RM) having three groups and six classes, which can prove very useful for the pearl millet breeders to develop low rancid pearl millet lines (Figure 5).

**Figure 6:**
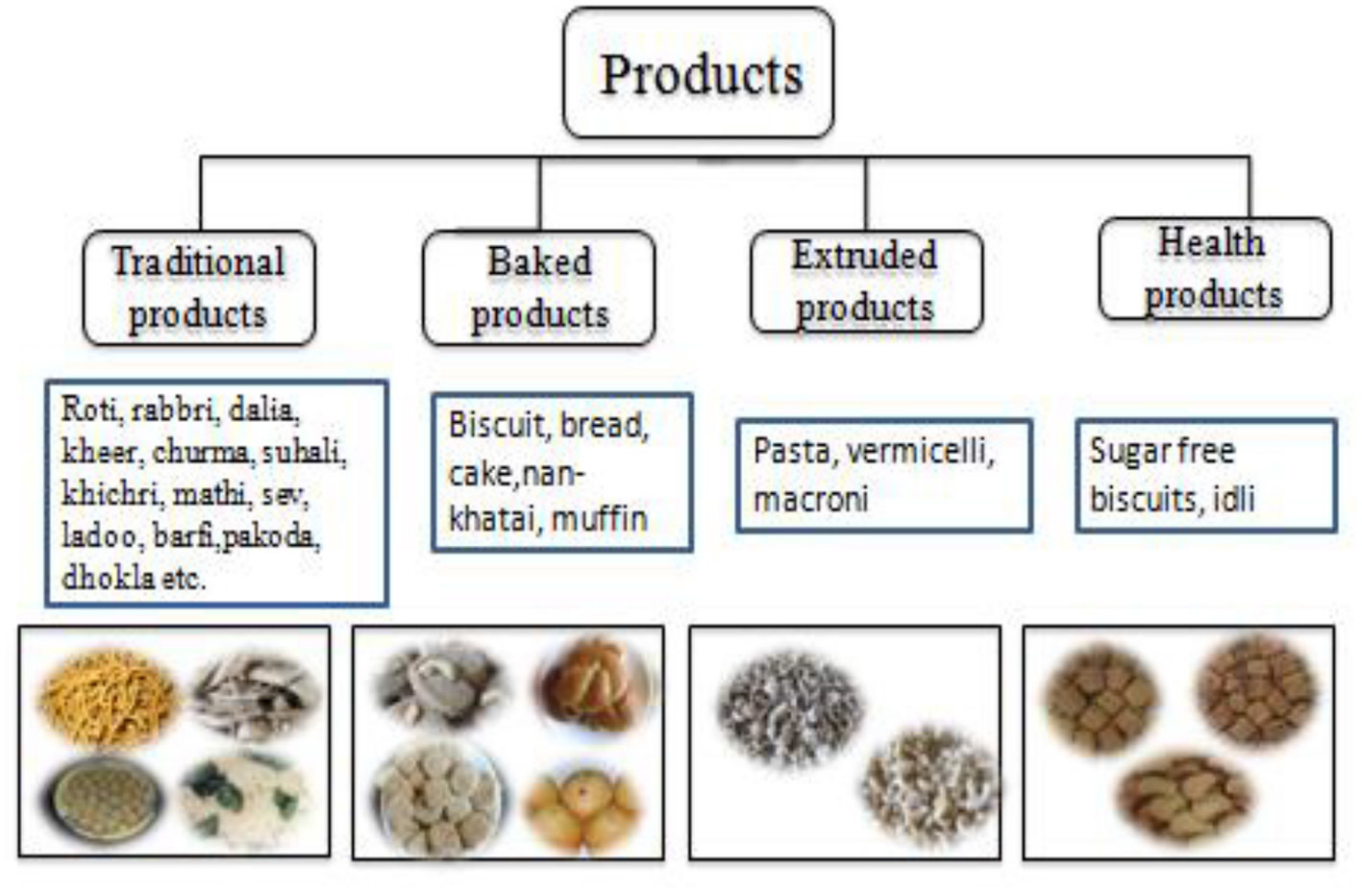
Delicious and value-added food products from pearl millet.

Pearl millet is used in different forms at a global level: unleavened bread (roti or chapatti), porridge, gruel, dessert etc. and it is generally defined as a poor man's bread. Its flour can substitute (10–20%) for wheat flour in “whole-grain” breads, pretzels, crackers, tortillas and dry and creamed cereals (Dahlberg et al., 2003). In India and Africa, it is primarily grown for food and forage, while in the American continents, it is a main component of poultry and livestock sector (Serba et al., 2020). Value addition is also very useful to promote its consumption, and several value-added products are gaining popularity among people. It can be used for making various traditional food products such as khichri, roti, sakarpare, gulgule etc., while industries are also using it for making products such as noodles, pasta, vermicelli, biscuits, bread, cookies, cakes, puffs etc in India (Figure 6). Several indigenous foods and drinks are made from flour/meal and malt of the millet in Africa and are nutritionally superior to other cereals. They contain high protein (up to 9.5/100 g for teff and fonio), ash, calcium (up to 344 mg/100 g for finger millet), phosphorus and potassium (up to 250 mg/100 g), iron and zinc levels (Obilana and Manyasa, 2002). The main food items prepared from pearl millet vary in the different countries of West Africa. The stiff or thick porridges (Tuwo or Tộ) are very famous and generally used in all the Sahelian countries, while steam-cooked product “Couscous” is more common in the Francophone countries, including Senegal, Mali, Guinea, Burkina Faso, Niger, and Chad. The thin porridge “bouillie” is also popular in these countries. In Nigeria and Niger, the thin porridge “Fourra” and *Masa*, a fried cake are very popular, while “Soungouf,” “Sankhal” and “Araw” are mainly popular in Senegal (Kaur et al., 2014; Ajeigbe et al., 2020). Its grains are also locally brewed to produce non-alcoholic or alcoholic beverages in Asia and Africa (Dwivedi et al., 2012).

Among the 26 major risk factors for the disease estimates at the global level, Fe deficiency positions at ninth and Zn deficiency falls at 11^th^ position (Ezzati et al., 2002). This issue is mainly severe in developing countries and infants, pregnant women, and adolescent children are particularly among high-risk groups. In India, ~80% pregnant women, 52% non-pregnant women and 74% of children lying in the age group of 6–35 months are suffering from anemia caused by Fe deficiency (Kramer and Allen, 2015). On the other hand, 50% of the world population is affected by zinc (Zn) deficiency, ultimately leading to diarrhea, impaired physical growth, and suppressed immune system (Gibson et al., 2008). In this context, biofortification of staple crops is a good, cost-effective, and sustainable approach to address malnutrition caused by micronutrients (Stein et al., 2007; Bouis et al., 2011). Biofortification is a multidisciplinary approach that can utilize the full potential of crop improvement and nutrition science and eliminate the prolonged issue of micronutrient malnutrition. Recent study showed that consuming biofortified pearl millet at 250 g day^−1^ can meet 84% of the RDA for iron and 100% of the RDA for zinc in non-pregnant non-lactating (NPNL) women, while ordinary pearl millet can fulfill only 20% of their iron requirement (Neeraja et al., 2017). Absorption or bioavailability of iron in pearl millet is enough to meet out >50% of the daily requirement of adult males or children. In pearl millet, the bioavailability of Fe can be assumed to be 7.0–7.5% (Govindaraj et al., 2019). Approximately 50–100% of the daily requirement of iron can be met out with one meal of biofortified high-iron variety and is sufficient enough to overcome deficiency of iron in children, women and men (Kodkany et al., 2013; Mahendrakar et al., 2019).

Pearl millet as such is already a high-iron crop along with high Zn content; however, commercially available cultivars have lower Fe and Zn contents. Biofortification priority index (BPI) indicates that pearl millet is a major target crop for iron and zinc biofortification. Enormous genetic variability (30–140 mg/kg Fe and 20–90 mg/kg Zn) can be used efficiently for developing high-yielding cultivars along with high iron and zinc densities (Kanatti et al., 2014). Targeting the development of biofortified crops with enhanced nutrients, ICAR is supporting a Consortia Research Platform (CRP) of rice, wheat, maize, sorghum, pearl millet and small millets since 2014. Several promising donors have been identified and breeding material is being generated combining high nutrient content and yield. Thus, focus was also laid on nutritional improvement in addition to yield improvement in pearl millet. Development of Fe and Zn-biofortified varieties/hybrids was considered as a high-priority area in pearl millet resulting in the inclusion of biofortification in the main stream and now it is a routine affair. The gradual shift of efforts of biofortification research from exploring variability in 2008 to breeding high-Fe cultivars by exploiting existing high-Fe lines in 2011 and testing and delivery of biofortified varieties and hybrids was recognized in India and became conscious breeding part in pearl millet at various centers of public and private partners. The first high-iron pearl millet variety “Dhanashakti” was released for the state of Maharashtra in 2013, and subsequently, it was notified and released by Central Variety Releasing Committee in April 2014, for growing in all pearl millet-growing regions of India. Inclusion of nutritional quality traits for Fe (42 ppm) and Zn (32 ppm) in the varietal promotion criteria is first of its kind in any of the food crop and the world too (Satyavathi, 2017). Since then, many hybrids/varieties were developed as micronutrient-rich pearl millet cultivars, including the seven biofortified cultivars identified from the special biofortification trial. In addition, a project on “Development of Biochemical and Physical Processing Technology to Arrest Oxidation of Lipids/Flavones to Enhance the Shelf-Life of Pearl Millet Flour” under Niche area of excellence (NAE) program has been started in ICAR-IARI, India, to overcome the issue of rancidity and enhance shelf life of pearl millet flour. Parallel research programs addressing the issues of rancidity were also taken up in ICRISAT along with ICAR.

Screening of enormous genetic materials such as germplasm collections, elite lines, hybrids, segregating populations etc. as well as phenotyping for iron and zinc through destructive techniques and breeding resources is required to breed micronutrient dense cultivars. Several studies have been conducted by several research groups around the world to assess the genetic diversity for Fe and Zn contents from time to time using different sets of germplasm accessions and breeding lines (Velu et al., 2007; Rai et al., 2012, 2014; Kanatti et al., 2014; Anuradha et al., 2017; Kumar et al., 2018; Govindaraj et al., 2019, 2020). Various studies were reported to show a significant importance of environment and G × E interactions to determine the levels of grain Fe and grain Zn contents in pearl millet. Such studies also identified donors that are high and stable micronutrient contents (Satyavathi et al., 2015; Rai et al., 2016; Pawar et al., 2018; Singhal et al., 2018). In addition, many efforts were put to discover, validate and deploy trait-based molecular markers for iron and zinc contents in pearl millet (Kumar et al., 2016, 2018; Anuradha et al., 2017). Manwaring et al. (2016) elaborated the several challenges and opportunities associated with biofortification of pearl millet and emphasized that naturally occurring genetic variations existing in germplasm collections should be incorporated into elite, micronutrient-rich varieties through different advanced platforms to develop bifortified varieties.

**Figure 7:**
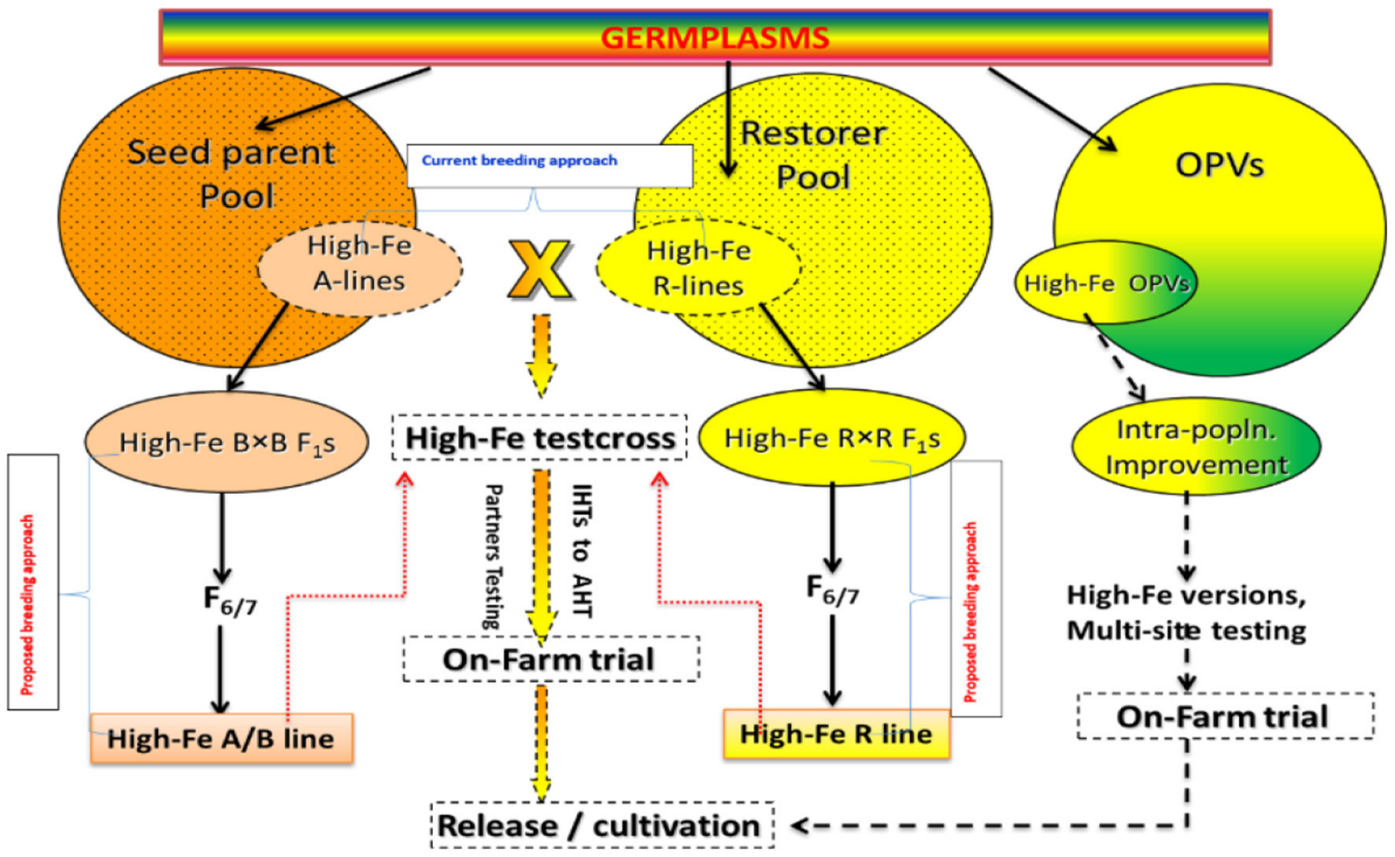
Current breeding approaches followed for developing biofortified cultivars in pearl millet (Adapted from: Govindaraj et al., 2019).

The success of pearl millet biofortification program lies in the high-throughput precision phenotyping for the estimation of grain micronutrient content. Further, demonstration of the feasibility of developing high-Fe and high-yielding hybrids and encouraging the partners to breed for these micronutrient traits by means of mainstreaming the biofortification can be an important approach for its promotion. There is a high need to facilitate the focused screening of partners breeding materials for grain Fe and Zn contents for their introgression of high Fe content into locally adapted, high-yielding and farmer-preferred cultivars (Figure 7). Promotion and strengthening of the pipeline of high-iron and high-yielding partners-bred hybrids and testing of their grain samples for grain Fe and Zn contents can be a useful step for enhancing its importance. In addition to this, bioavailability is another aspect that needs to be focused to get full potential of this crop.

## Challenges for Pearl Millet

Despite the breeding efforts, most of breeding programs fail to deliver hybrids due to a vast variation in microclimate (day and night temperature and humidity) and soil apart from rainfall, which requires proper quantification. Further, narrow cultivar diversity in drought-prone ecology also is another factor for this. Thus, there is a high need to give higher priority to the below-mentioned areas to promote its production and utilization:

- Development of hybrids/varieties of pearl millet with better regenerative capacity on reversal of dry spell for harsh environment/drought-prone areas (for A_1_ zone in India).
- Development of hybrids/varieties resistant/tolerant to salt/high temperature.
- Shift in focus of breeding from productivity improvement to the identification of end product-specific traits.
- Mainstreaming of biofortification in pearl millet for iron and zinc.
- Enhancement of shelf life of pearl millet flour and overcome rancidity to promote its products.
- Development of screening protocols and control measures against different diseases such as downy mildew, blast, rust, ergot, smut.
- Generating authentic data on nutritional benefits of pearl millet and bioavailability studies.
- A study on demand survey for pearl millet.

## Conclusion

Pearl millet being a climate-resilient crop along with high nutritional value can be exploited for improving nutritional quality and combating malnutrition. It is almost free from major diseases and insect attacks and could be cultivated with a good harvest. Hence, the focus should be laid towards the development of food products from pearl millet to make it acceptable as an alternative crop of the future. Indian policymakers need to refocus their interest toward millet farming systems and policies should be engraved for creating a feasible environment for pearl millet farmers. After the successful use of genomic tools, screening and development of improved genotypes have become easy and fast, and the progress toward enhancing the use of genomic resources is quite appreciable. But it can be characterized further for harnessing the natural genetic variation within the germplasm, and a lot of efforts are still required to execute genomics to improve the crop using high-throughput genome analysis, sequence-based molecular markers, NGS techniques and genome editing etc. The genomics and breeding platform needs a better alignment and constant up-gradation to develop improved hybrid parental lines, and populations must be adapted specifically according to the specific global agro-ecologies. The idea of genetic gains, genome editing, pre-breeding, and speed breeding can be also very useful for the researchers in selecting plants for desired traits along with many variations. On the other hand, the construction of high-density maps, QTL detection, candidate gene identification, new genome sequence techniques, use of advanced multi-parental and AM panels, GWAS and GS can speed up the recognition of allelic variants for pearl millet improvement. Mapping of several abiotic stresses, QTLs, etc. is highly desired to combat global climatic effects and the recently developed technologies must be tested under actual conditions.

The outcomes from model crops can be used in pearl millet to achieve added improvement and develop Zn- and Fe–enriched biofortified varieties. Synteny studies can prove useful for the identification of common genes linked with nutrition biosynthesis pathways, and these should be incorporated into pearl millet by traditional breeding or transgene techniques for further nutritional improvements. In addition, nutritional as well as the economic security of small and marginal farmers, enhancing demand of pearl millet, value addition and market-led extension through food science and nutrition is vital to promote the cultivation and consumption of this crop. In conclusion, multidisciplinary approaches, including breeding, genomics, bioinformatics, biotechnology, nutrition and genetics etc. are required to exploit and harness the beneficial attributes of nutricereal pearl millet for combating changing climate and attaining nutritional security.

## Author Contributions

CTS has conceived the idea of writing this review, drafted and edited the manuscript. SA contributed in writing and editing the manuscript. RKS and VK contributed in manuscript preparation.

## Conflict of Interest

The authors declare that the research was conducted in the absence of any commercial or financial relationships that could be construed as a potential conflict of interest.

## Publisher's Note

All claims expressed in this article are solely those of the authors and do not necessarily represent those of their affiliated organizations, or those of the publisher, the editors and the reviewers. Any product that may be evaluated in this article, or claim that may be made by its manufacturer, is not guaranteed or endorsed by the publisher.
